# Creating Inclusive Design Experiences Through Engaging Seniors With Disabilities in Student Hackathons

**DOI:** 10.1093/geroni/igaa057.2021

**Published:** 2020-12-16

**Authors:** Jon Sanford

**Affiliations:** Georgia Tech, Atlanta, Georgia, United States

## Abstract

Although voice-activated assistants (e.g., Amazon Alexa) have become smarter, faster, more personalized, and more ubiquitous, little is known about their potential to promote aging in place for people with disabilities. Partnering with Amazon’s Alexa team, a 2-month long design competition and hackathon was conducted to inspire college students to develop innovative voice-activated solutions to support successful aging with disabilities. This presentation will cover the specific inclusive experiences used to immerse student teams in the daily lives of the target population to ensure that design solutions responded to real needs of real people in real environments. These included: lectures on current research findings about the everyday needs and challenges of the target users as well as universal design approaches to solving those problems; a survey of individuals currently using voice-activated assistants to understand their benefits and potential uses; and providing target with Alexa-enabled devices and embedding them into the hackathon teams.

